# An integrated surveillance in Zhejiang Province: ecological and pathogen survey of vectors and reservoir hosts in 2024

**DOI:** 10.3389/fvets.2025.1713567

**Published:** 2026-02-02

**Authors:** Jinna Wang, Mingyu Luo, Qinmei Liu, Tianqi Li, Zhou Guan, Zhenyu Gong, Juan Hou, Jimin Sun, Jianmin Jiang

**Affiliations:** Zhejiang Provincial Center for Disease Control and Prevention, Hangzhou, China

**Keywords:** ecological surveillance, etiology surveillance, host, integrated surveillance, vector, vector-borne disease

## Abstract

**Introduction:**

In this study, an integrated surveillance framework was employed to simultaneously quantify the population densities of key vectors and reservoir hosts and screen them for associated pathogens across Zhejiang Province, China.

**Methods:**

The light trap method, larval pipette method, fly trap method, sticky trap method, trap-night method, tick-picking method, dragging method, visual inspection method, and chigger mite picking method were used for the ecological surveillance of mosquitoes, flies, cockroaches, rodents, ticks, bedbugs, and chigger mites. Rodent samples were screened for Dabie Bandavirus, Hantavirus, *Leptospira interrogans*, *Rickettsia typhi*, and *Orientia tsutsugamushi*. Mosquito samples were tested for dengue, yellow fever, Japanese encephalitis, West Nile, Zika, and chikungunya viruses. The descriptive statistics were used for analysis.

**Results:**

In 2024, the mosquito density in Zhejiang Province was 16.03 mosquitoes per trap-night, with *Culex tritaeniorhynchus* (59.05%) and *Culex pipiens pallens* (31.37%) being the dominant species. Livestock sheds harbored the greatest mosquito densities, averaging 81.07 mosquitoes per trap-night. The average BI was 12.97. The rodent density was 0.34 rodents per 100 trap-nights, and the dominant species was *Rattus norvegicus.* The fly density was 3.06 flies per trap, with Sarcophagidae species being dominant. The cockroach density was 0.44 cockroaches per trap, with *Blattella germanica* comprising 97.13% of the total catch. The tick densities were 0.51 ticks per animal and 0.35 ticks per flag per 100 m. No bedbugs were detected. The chigger mite infestation rate was 71.11%. Regarding rodent-borne pathogens, the positivity rates for Hantavirus, *L. interrogans*, and *O. tsutsugamushi* were 2.42, 10.46, and 0.16%, respectively. No Dabie Bandavirus or *R. typhi* were recorded. All 27,402 mosquitoes tested negative for the target pathogens.

**Conclusion:**

This integrated surveillance established baseline metrics for important vectors and reservoir hosts, furnishing evidence-based support for the ongoing management and prevention of vector-borne diseases.

## 1 Introduction

Vector-borne diseases (VBDs) are caused by pathogens that are transmitted to humans by hematophagous arthropods, including mosquitoes, flies, lice, and ticks, among others. VBDs such as Zika, Dengue, West Nile fever, Chikungunya, and Yellow fever remain major global public health threats (1). Estimates suggest that more than half of the world’s population lives in areas where at least two VBDs are present (2). Furthermore, VBDs account for 17% of the global estimated burden attributable to infectious diseases (3). Vector-borne diseases are typically not directly transmissible between humans. Instead, they are mainly spread under suitable conditions involving interactions among vectors, animal hosts, the environment, pathogens, and vulnerable human populations (1).

The global incidence of VBDs is rising, fueled by climate change and increasing human-vector contact. For instance, dengue cases increased nearly 450% globally from 1990 to 2013 (4), resulting in a substantial disease burden. Beyond pathogen transmission, vectors and their animal hosts can also directly cause discomfort, allergic reactions, skin lesions, and pain in humans (5). Given the current lack of effective medication or vaccines for some VBDs, the control of vectors and host animals has traditionally been the primary method for the prevention of VBDs, and remains highly effective (6). Moreover, for several diseases, it constitutes the only available control tool. Consequently, implementing control measures against vectors and host animals before they can transmit pathogens to humans remains the most effective strategy for preventing VBDs.

Continuous surveillance of vector and host animal abundance, seasonality, and infection status is essential for identifying dominant species, peak-risk periods, and local transmission hotspots. Different types of vector or host monitoring systems have been established worldwide (7–9) and play an important role in VBD prevention. However, most surveillance programs remain single-species focused, addressing either vector ecology or pathogen carriage in isolation. The One Health concept has recently transformed VBD surveillance by unifying animal, human, and environmental health perspectives, thereby creating an integrated framework that is now central to effective VBD prevention and control (10, 11).

Semenza et al. claimed the urgent action for integrated surveillance for VBDs in Europe (12). Their findings indicated that the monitoring of environmental and climatic precursors of VBDs, combined with the integrated surveillance of human cases and vectors, could help counteract potential impacts and enhance early warning and response capabilities. An integrated surveillance system had also been tested in pilot programs in Zhejiang Province, China, and resulted in a 21.6% cost saving in rodent-borne disease surveillance (13). Consequently, integrated surveillance has become a cornerstone technology for the monitoring and control of VBDs.

Zhejiang Province, located on the southeast coast of China, features a complex topography of plains, hills, and mountains combined with a subtropical monsoon climate characterized by high humidity and abundant rainfall. These conditions create extensive vector breeding habitats. Densely populated urban and rural settlements, coupled with large-scale population movement from tourism and trade have contributed to the province’s high vector-borne disease burden (14, 15). Accordingly, an integrated province-wide surveillance program was launched in 2024 to quantify the abundance, species composition, seasonal dynamics, and infection status of key vectors and reservoir hosts throughout the province. The surveillance mainly encompassed monitoring of mosquitoes, flies, rodents, cockroaches, ticks, chigger mites, and bed bugs, along with pathogen detection in mosquitoes and rodents. The surveillance findings provide evidence-based support for the prevention, control, and early warning of VBDs across Zhejiang Province.

## 2 Materials and methods

### 2.1 Study design

**Figure 1:**
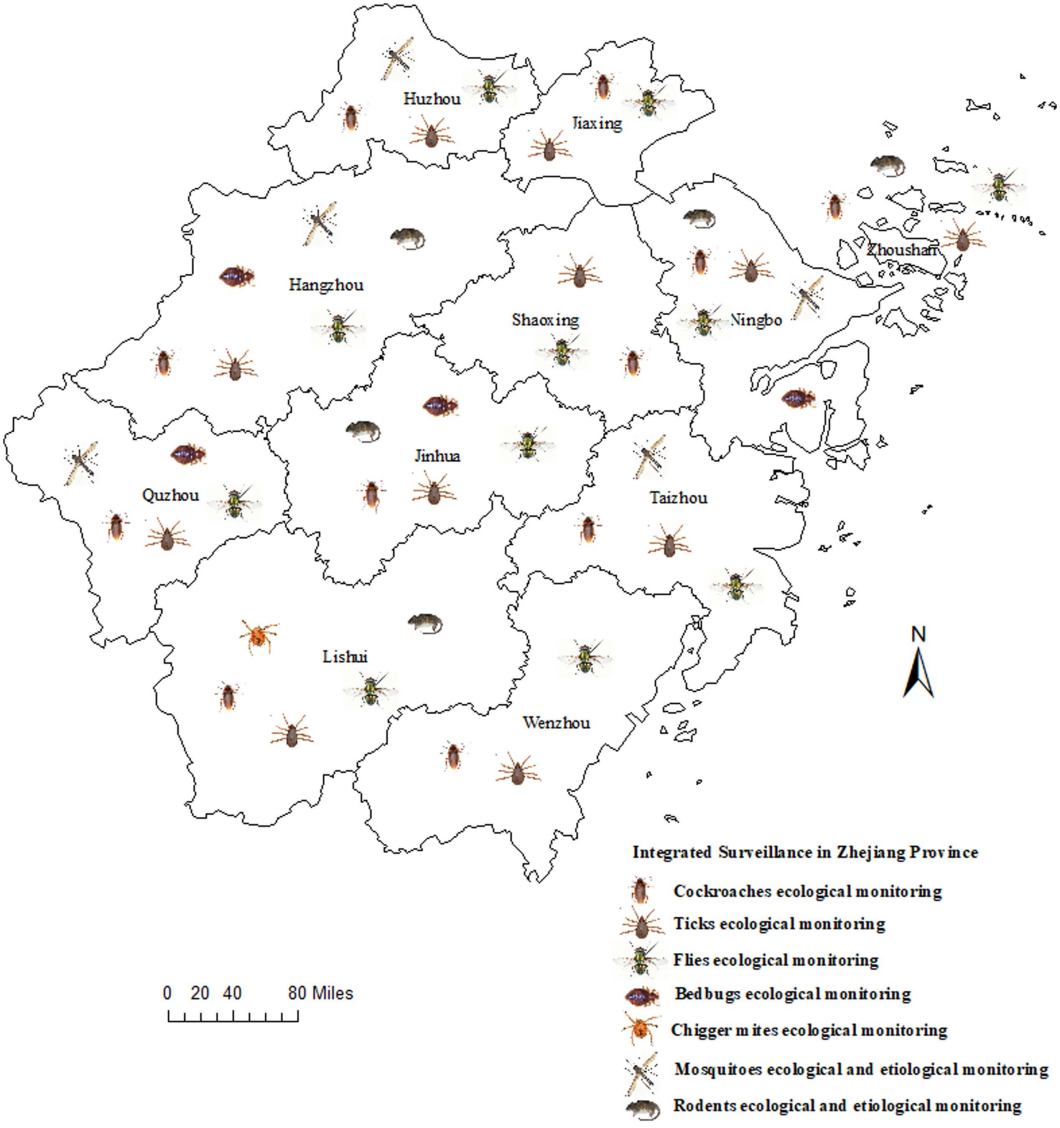
The regional distribution of the integrated surveillance in Zhejiang Province in 2024.

The integrated surveillance conducted in Zhejiang Province included the monitoring of mosquitoes, flies, rodents, cockroaches, ticks, chigger mites, and bedbugs, in addition to a pathogen survey of mosquitoes and rodents. All 11 prefecture-level cities within Zhejiang Province—Hangzhou, Ningbo, Wenzhou, Huzhou, Jiaxing, Shaoxing, Jinhua, Quzhou, Lishui, Taizhou, and Zhoushan—were included in the study in 2024 (Figure 1). Monitoring sites for different vectors or host animals were selected based on habitat type and related diseases. For the surveillance of mosquitoes, flies, rodents, and cockroaches, all districts and counties within each selected prefecture-level city were included in the monitoring. For ticks, chigger mites, and bedbugs, at least one county or district was selected for each prefecture-level city. All collected vectors were delivered to local municipal Centers for Disease Control and Prevention (CDC) laboratories under cold-chain conditions. All species were identified using a stereomicroscope based on morphological characters using dichotomous key from *The Identification Pictures of Common Medical Vectors in Chinese Ports* (16) or detection images (VHX-7000 Digital Microscope, KEYENCE). Adult mosquito and rodent samples were screening for pathogens.

### 2.2 Mosquito surveillance

Mosquito surveillance timing was based on local climatic conditions and seasonal vector population dynamics. Due to low winter temperatures in Zhejiang Province that result in negligible mosquito densities, monitoring was conducted monthly from April to November 2024. Adult mosquito density was monitored using the light trap method. At least two sites were selected for each of the five habitats, including urban residential areas, parks, hospitals, rural residential houses, and livestock sheds in each county or district. Locations far from interfering light sources and sheltered from the wind were chosen as the surveillance sites. The light traps were placed 1.5 m above the ground. In each county, at least 10 light traps were turned on 1 hour before sunset, and remained on until 1 hour after sunrise the next day. The captured mosquitoes were identified and counted in the laboratory. For mosquito larvae monitoring, the larval pipette method was used in all county-level administrative divisions once a month from April to November in 2024. For each monitoring county, four neighborhoods or villages situated in different geographical directions were selected, with no fewer than 100 households investigated per county. All containers in or near the houses were examined for the presence of *Aedes* larvae, including discarded tires, natural water accumulations (bamboo tubes, tree holes, stone cavities, etc.), idle containers, water storage tanks, small puddles in green belts, bonsai and aquatic plant containers, artificial rock pools, and other potential water-holding containers.

### 2.3 Fly and cockroach surveillance

Flies were monitored using the fly trap method once a month from April to November. In each county or district, two sites were selected for each of four specified habitats: farmers’ markets, urban residential areas, green spaces, and the outdoor environments of catering areas. In each county, at least eight fly traps were placed before 09:00 h on the first day, and were collected after approximately 24 h.

Cockroach density was monitored using the sticky trap method once in every second month in 2024. Two sites were selected from each of the following habitats: farmers’ markets, supermarkets, catering establishments, hotels, urban residential areas, and hospitals. At least 10 cockroach sticky traps, containing 2 grams of fresh breadcrumbs in the center as bait, were set out in each site in the evening and collected the next morning.

### 2.4 Rodent and chigger mite surveillance

Rodent monitoring was performed bimonthly throughout 2024 using the trap-night method. Urban residential areas, rural residential areas, and key industries were chosen as the habitats. At least 200 mousetraps were set per habitat during each monitoring session. Indoors, medium mousetraps (Size: 12 cm × 6.5 cm, medium-sized commercial mousetrap, Xiangyun County Hongjin Mousetrap Factory, China) were distributed to cover 15 m^2^. In rooms exceeding 100 m^2^, traps were placed every 5 m along the wall base. All traps were placed at dusk and retrieved the next morning.

Chigger mite surveillance was performed three times a year (May, July, and September) in 2024. The chigger mites were collected from the rodent’s body. The monitoring environments included farmlands, orchards, forests, wastelands, rural residential areas, urban residential areas, key locations, and parks. The surface of each rodent’s body was carefully examined for the presence of chigger mites.

### 2.5 Tick surveillance

Tick monitoring was undertaken in March, May, July, and September of 2024. Two sampling methods were used: tick-picking and flagging methods. Tick-picking method was used on animal body surfaces. At least 10 animals (e.g., sheep, cattle, and dogs) in rural areas were examined for ticks at each monitoring site and during each monitoring session. The questing ticks were sampled by flagging method. This involved slowly dragging a white or light-colored cotton flannel cloth flag measuring 90 × 60 cm through vegetation for at least 30 min. Ticks that attached to the flag were collected every 10 meters.

### 2.6 Bedbug surveillance

Bedbug surveillance was conducted twice a year using the visual inspection method. The habitats for bedbug monitoring included collective dormitories (in factories, schools, etc.), hotels, nursing homes, cinemas, transportation vehicles, and urban residential areas, among others. Areas where bedbugs were likely to be present, such as bed boards, bed frames, mattresses, wall cracks, tables, chairs, and sofas, were searched for signs of bedbug infestation, including fecal stains, bloodstains, exuvias, eggs, dead bedbugs, and live bedbugs. A minimum of 30 units were surveyed in each monitored district or county, with each unit containing at least 10 standard rooms.

### 2.7 Rodent pathogen screening

Most rodent samples used for pathogen detection were obtained during the 2024 density surveillance, with supplementary trapping undertaken in each city to ensure a minimum sample size of 200 animals. Captured rodents were placed in sealed bags with their traps and transferred to an airtight container for diethyl ether fumigation (≥10 min) in the laboratory. Then the dead rodents were dissected in the laboratory, and the liver, spleen, lungs, and kidneys were collected. All the samples were stored at −70 °C before testing. Dabie Bandavirus was assayed in the liver, spleen, and lung; the assay for Hantavirus was performed in the lung; and assays for *Leptospira interrogans* (Stimson, 1907) Wenyon (42), *Rickettsia typhi* (43) Philip, 1943, and *Orientia tsutsugamushi* (Hayashi, 1920) Tamura et al. (57) were conducted in the liver, spleen, and kidneys of the rodents. The samples were homogenized and centrifuged. 25–30 mg of rodent samples were excised and placed in a 2 mL centrifuge tube containing 20–30 grinding beads (Φ1.2 mm) and 200–300 μL of 1 × PBS buffer. Homogenization was performed automatically using Precellys 24 Bead Mill (Bertin Technologies, France) with the following program parameters: speed 1,600 rpm, duration 30 s, pause interval 10 s, repeated for 3 cycles. After brief centrifugation, it was used for subsequent experiments. Then total RNA and DNA were extracted automatically from the homogenates using a Magnetic Viral DNA/RNA Fast Kit (TIANGEN, China) by TGuide S32 Nucleic Acid Extractor (TIANGEN, China) according to the manufacturer’s instructions. Real-time quantitative fluorescence PCR assays were then performed using the Takara PrimeScript One Step RT-PCR Kit (Takara, Japan). The total volume of the reaction system of hantavirus and Dabie Bandavirus was 25 μL, including 2 × Reaction Mix 12.5 μL, probe (10 μM) 0.3 μL, upstream and downstream primers (10 μM) 0.5 μL, RNA template 5 μL, Enzyme mix 1.0 μL, and deionized water supplement. The cycling parameters included 50 °C for 30 min (one cycle), 95 °C for 10 min (one cycle), 95 °C for 15 s and 60 °C for 45 s (40 cycles). For the *L. interrogans*, *R. typhi* and *O. tsutsugamushi*, the total volume of the reaction system was 20 μL, which included Taq DNA polymerase and dNTP mixture qPCR Master Mix 10 μL, probe 0.4 μL (final concentration 200 nmol/L), upstream and downstream primers 0.8 μL (final concentration 400 nmol/L), DNA template 3–5 μL, deionized water complement. The cycling parameters included denaturation at 95 °C for 5 min (one cycle), amplification at 95 °C for 15 s and 60 °C for 45 s (40 cycles). Target gene sequences were provided by the China CDC, and were also referenced in Wang et al.’s study (17) (Table 1). Data were analyzed using the manufacturer’s software. The cycle threshold (Ct) values were set as ≤35 for *L. interrogans*, Hantavirus, and Dabie Bandavirus; ≤36 for *R. typhi*; and ≤33 for *O. tsutsugamushi*.

**Table 1:** Primers and probes for the rodents pathogens screening.

Pathogens	Primers and probes	Sequences (5′- 3′)
*Orientia tsutsugamushi*	Ot56 kD-F	CGCCAGTRATMATTCCTCCRA
	Ot56 kD-R	TTTYWGCTAGTGCRATAGAATTRG
	Taqman-Ot	FAM-TAAGGACCACACTCTAATC-MGB
*Leptospira interrogans*	Lepto F	CCCGCGTCCGATTAG
	Lepto R	TCCATTGTGGCCGR(A/G)ACAC
	Lepto P	FAM-CTCACCAAGGCGACGATCGGTAGC-BHQ
Hantavirus (HTNV)	F(771–793)	GCTTCTTCCAGATACAGCAGCAG
	R(862–884)	GCCTTTGACTCCTTTGTCTCCAT
	P(811–839)	CCTGCAACAAACAGGGAYTACTTACGGCA
Hantavirus (SEOV)	F(217–237)	GATGAACTGAAGCGCCAACTT
	R(272–291)	CCCTGTAGGATCCCGGTCTT
	P(239–263)	CCGACAGGATTGCAGCAGGGAAGAA
Dabie Bandavirus (S gene)	S-F-3	GGGTCCCTGAAGGAGTTGTAAA
	S-R-3	TGCCTTCACCAAGACTATCAATGT
	S-Probe-3	TexasRed-TTCTGTCTTGCTGGCTCCGCGC-BHQ-2
Dabie Bandavirus (L gene)	L-F-3	AGTCTAGGTCATCTGATCCGTTYAG
	L-R-3	TGTAAGTTCGCCCTTTGTCCAT
	L-Probe-3	HEX-CAATGACAGACGCCTTCCATGGTAATAGGG-BHQ1
Dabie Bandavirus (M gene)	M-F-3	AAGAAGTGGCTGTTCATCATTATTG
	M-R-3	GCCTTAAGGACATTGGTGAGTA
	M-Probe-3	FAM-TCATCCTCCTTGGATATGCAGGCCTCA-BHQ-2
*Rickettsia typhi*	Pr47F	TGTTGATGGTGCAGGATTTGA
	Pr110R	CGAATTTGTAGCGACAGGAAGA
	mo-T	FAM-CAAACTGGCGCTGGTGT-MGB

### 2.8 Mosquito pathogen screening

For mosquito-borne pathogen screening, adult mosquito specimens obtained through routine surveillance were supplemented with additional trapping to reach the required sample size. In each city, at least two mosquito species were targeted for pathogen detection, with a minimum of 1,000 adult females tested per species. The mosquitoes were stored at −70 °C in the laboratory before detection. All sampled mosquitoes were pooled according to species, habitat, and date, with a maximum of 20–30 individuals per pool. The samples were placed in a 2 mL centrifuge tube containing an appropriate amount of zirconia beads (Φ1.2 mm, 20–30 beads) and 0.5–1 mL of Hank’s solution. Homogenized using Precellys 24 Bead Mill (Bertin Technologies, France) until complete tissue disruption was achieved, briefly centrifuged, and then used for nucleic acid extraction. The RNA/DNA was extracted automatically from the mosquitoes using the Magnetic Viral DNA/RNA Fast Kit (TIANGEN, China) by TGuide S32 Nucleic Acid Extractor (TIANGEN, China) according to the manufacturer’s protocol. The PrimeScript One Step RT-PCR Kit version 2 (Takara, Japan) was used for reverse transcription. The cDNA product from the first round of amplification served as a template for the second round of amplification using Takara Ex Taq version 2.0 plus dye (Takara, Japan). The sequences of the target genes were provided by the China CDC, and were also referenced in Wu et al.’s study (18). The total volume of the reaction system was 25 μL. Primer sequences, amplification fragments, and other information were listed in Table 2. Finally, the PCR products were analyzed by 1.2% agarose gel electrophoresis using GelRed Nucleic Acid Gel Stain (10,000×, Biontium) and imaged using a Shanghai QINXIANG Gel Imager (model: GenoSens 2,200). Pathogen testing for *Aedes albopictus* (44) included dengue virus, yellow fever virus, Japanese encephalitis virus, West Nile virus, Zika virus, and chikungunya virus; for *Culex tritaeniorhynchus* Giles (45), the pathogens assayed included Japanese encephalitis virus, West Nile virus, Zika virus, and chikungunya virus; for *Culex pipiens pallens* Coquillett (46), the pathogens tested were Japanese encephalitis virus and West Nile virus; and for *Anopheles sinensis* Wiedemann (47), the pathogen tested was Japanese encephalitis virus.

**Table 2:** Primers and reaction conditions for testing *Flavivirus* and *Alphavirus* in mosquitoes.

Pathogens	PCR amplification	Primers	Sequences (5′- 3′)	Fragment size (bp)	Reaction conditions
*Flavivirus* genus	Round 1	XF-F1	AACATGATGGGVAARMGWGARAA	263	95°C for 10 min; 95°C for 30 s, 52 °C for 30 s, 72 °C for 30 s (35 cycle); 72 °C for 5 min.
		XF-R	GTRTCCCANCCDGCDGTRTCATCNGC		
	Round 2	XF-F2	AARGGMAGYMGNGCHATHTGGT	215	95°C for 10 min; 95 °C for 30 s, 54 °C for 30 s, 72 °C for 30 s (35 cycle); 72 °C for 5 min.
		XF-R	GTRTCCCANCCDGCDGTRTCATCNGC		
*Alphavirus* genus	Round 1	XA-F1	AGAGCRTTYTCGCATCTRGCYAK	433	95 °C for 10 min; 95 °C for 30 s, 54 °C for 30 s, 72 °C for 30 s (35 cycle); 72 °C for 5 min.
		XA-R	ACATGAACKGRGTKGTGTCRAASCCWAYCC		
	Round 2	XA-F2	TGCCCBRTGCGBAGYSCVGAAGAYCC	310	95 °C for 10 min; 95 °C for 30 s, 60 °C for 30 s, 72 °C for 30 s (35 cycle); 72 °C for 5 min.
		XA-R	ACATGAACKGRGTKGTGTCRAASCCWAYCC		

### 2.9 Statistical analysis

Ecological and etiological surveillance data for Zhejiang Province in 2024 were summarized primarily using descriptive statistics. All the descriptive statistics and plots were generated in R version 4.0.2 (The R Foundation for Statistical Computing) employing the ggmap package. The descriptive statistics primarily involved the density, species composition, and pathogen carriage rates of vectors and host animals. The adult mosquito density was calculated as the number of female mosquitoes caught per light trap per night. The Breteau index (BI) was calculated as the number of *Aedes* larvae-positive containers detected per 100 households (19). The fly density was calculated as the number of flies captured per trap. The cockroach density was calculated as the average number of cockroaches caught per sticky trap. The rodent density was calculated as the average number of rodents captured per 100 trap-nights. The ticks sampled on animals was calculated as the average number of ticks collected per animal examined. The density of questing ticks was calculated as the number of ticks caught per flag per 100 m of vegetation surveyed. The bedbug infestation rate was calculated as the number of rooms found to be positive for bedbugs per 100 rooms inspected. The rate of chigger mite infection was calculated as the average number of infected rodents found per 100 rodents inspected. The chigger mite index was calculated as the average number of chigger mites carried by each rodent. Positivity rate was calculated as the number of rodent or mosquito specimens testing positive for a specific pathogen divided by the total number of specimens tested. Average density was calculated as total captures divided by total sampling effort for each vector group.

## 3 Results

### 3.1 Mosquito density surveillance

A total of 73,550 households were surveyed for *Ae. albopictus* larvae in 2024, involving 47,284 containers, of which 9,539 were found to be positive for the larvae. The average BI was 12.97 ± 2.39. The maximum larvae density peak occurred in June and July, with BI values of 15.61 and 15.84, respectively. Among the cities assessed, Taizhou had the highest BI (16.91), followed by Quzhou (14.34), Ningbo (14.12), and Huzhou (13.13). The lowest BI (9.54) was recorded in Hangzhou. Among the different types of water bodies examined, the highest positivity rate was observed in discarded tires (35.26, 95% CI: 32.52–38.10%), followed by natural water accumulations (bamboo tubes, tree holes, stone cavities, etc.; 28.91, 95% CI: 23.21–35.36%), idle containers (22.34, 95% CI: 21.79–22.91%), water storage tanks (18.10, 95% CI: 17.44–18.78%), small puddles in green belts (18.09, 95% CI: 15.64–20.82%), bonsai and aquatic plants (15.57, 95% CI: 14.83–16.35%), and artificial rock pools (14.26, 95% CI: 11.49–17.55%) (Supplementary Table 1).

A total of 5,770 mosquito light traps were set up across the province in 2024, resulting to the capture of 92,476 female mosquitoes, with an average mosquito density of 16.03 ± 26.86 mosquitoes per trap-night. Mosquitoes of the *Culex* Linnaeus, 1758 genus were the dominant catch when the light-trap method was used, accounting for 90.42% of the total catch (*Cx. tritaeniorhynchus*: 59.05, 95% CI: 58.73–59.36%, and *Cx. pip. Pallens*: 31.37, 95% CI: 31.08–31.67%). The remaining catch included small numbers of *An. sinensis* (5.96, 95% CI: 5.81–6.12%)*, Ae. albopictus* (1.93, 95% CI: 1.85–2.02%), and *Armigeres obturbans* Walker (48) (1.33, 95% CI: 1.26–1.40%). Maximum adult mosquito density occurred between July and September, reaching 26.87–33.12 mosquitoes per trap-night. Among the various habitats monitored, the highest density—81.07 mosquitoes per trap-night—was found in livestock sheds, where the greatest number of *Cx. tritaeniorhynchus* and *Cx. pip. Pallens* were captured. In the other habitats assayed, the densities were all below six mosquitoes per trap-night (Table 3). Among the different cities, Taizhou had the highest mosquito density (70.95 per trap-night), followed by Huzhou, with 36.74. The mosquito densities were relatively low in Ningbo, Hangzhou, and Quzhou, with values of 5.01, 3.28, and 2.73 mosquitoes per trap-night, respectively.

**Table 3:** Surveillance results for adult mosquitoes in different habitats of Zhejiang Province in 2024.

Habitats	No. of light traps set	No. of female mosquitoes captured	Mosquito density (mosquitoes per trap-night)	Female mosquitoes of different species					
				*Culex pipiens pallens*	*Culex tritaeniorhynchus*	*Aedes albopictus*	*Anopheles sinensis*	*Armigeres obturbans*	Others
Urban residential areas	1,202	5,119	4.26	4,560	141	351	21	41	5
Parks	1,162	4,540	3.91	3,876	147	430	20	65	2
Hospitals	1,162	4,348	3.74	3,942	78	238	16	71	3
Rural residential houses	1,363	7,047	5.17	5,808	553	458	130	93	5
Livestock sheds	881	71,422	81.07	10,827	53,686	311	5,328	957	313
Total (all habitats)	5,770	92,476	16.03	29,013	54,605	1788	5,515	1,227	328
Species composition (%) of female mosquitoes	–	–	–	31.37	59.05	1.93	5.96	1.33	0.36

### 3.2 Rodent density surveillance

A total of 176,386 effective mouse traps were placed, leading to the capture of 595 rodents, representing a density of 0.34 ± 0.18 rodents per 100 trap-nights. The dominant rodent species was *Rattus norvegicus* (Berkenhout, 1769), accounting for 43.36% (95% CI: 39.43–47.37%) of the total, followed by *Suncus murinus* (Linnaeus, 1766) (19.50, 95% CI: 16.51–22.87%), *Mus musculus* Linnaeus, 1758 (12.27, 95% CI: 9.87–15.15%), and *R. flavipectus* (49) (11.76, 95% CI: 9.42–14.60%). Other species of rodents were also captured, but in smaller numbers (Table 4). Among the three major habitats, rural residential areas had the highest rodent density (0.42 rodents per 100 trap-nights), while urban residential areas had the lowest (0.27 rodents per 100 trap-nights). Rodents were active throughout the year in Zhejiang Province, with peak activity occurring in May and November, exhibiting a discreet bimodal distribution. Jinhua had the highest rodent density (0.65 rodents per 100 trap-nights), followed by Hangzhou (0.36 rodents per 100 trap-nights), Zhoushan (0.35 rodents per 100 trap-nights), and Lishui (0.30 rodents per 100 trap-nights); the lowest density was recorded in Ningbo (0.08 rodents per 100 trap-nights).

**Table 4:** Surveillance results for rodents in different habitats of Zhejiang Province in 2024.

Habitats	No. of mousetraps set	No. of rodents captured	Rodent density (rodents per 100 trap-nights)	Rodents of different species									
				*Rattus norvegicus*	*Suncus murinus*	*Mus musculus*	*Rattus flavipectus*	*Niviventer confucianus*	*Apodemus agrarius*	*Rattus losea*	*Niviventer fulvescens*	*Leopoldamys edwardsi*	Others
Urban residential areas	58,830	156	0.27	62	44	21	15	7	5	1	0	0	1
Rural residential areas	58,745	244	0.42	69	65	28	30	9	34	3	1	1	4
Key industries	58,811	195	0.33	127	7	24	25	3	1	5	1	0	2
Total (all habitats)	176,386	595	0.34	258	116	73	70	19	40	9	2	1	7
Species composition (%) of rodents	–	–	–	43.36	19.50	12.27	11.76	3.19	6.72	1.51	0.34	0.17	1.18

### 3.3 Fly density surveillance

**Figure 2:**
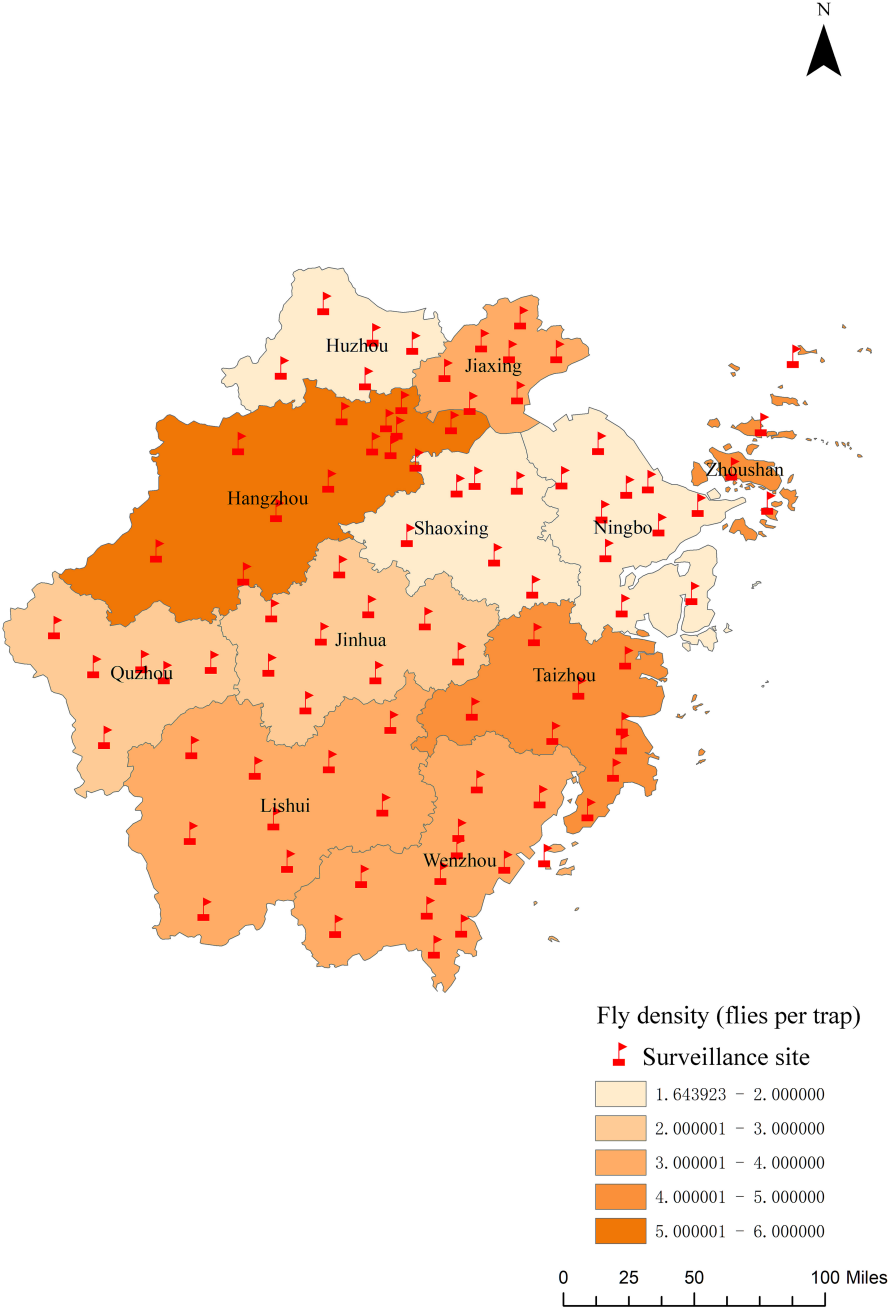
The regional distribution of fly density in Zhejiang Province in 2024.

A total of 6,751 fly traps were applied across the province in 2024, capturing 20,687 flies, with a density of 3.06 ± 1.18 flies per trap (Supplementary Table 2). Flies of the Sarcophagidae Haliday (50) family predominated, accounting for 32.93% (95% CI: 32.30–33.58%) of the total catch, followed by *Musca domestica* Linnaeus, 1758 (20.97, 95% CI: 20.42–21.53%), *Lucilia sericata* (51) (11.38, 95% CI: 10.96–11.82%), *M. stabulans* (52) (8.37, 95% CI: 8.00–8.76%), *M. sorbens* (53) (6.23, 95% CI: 5.90–6.56%), *Chrysomyia megacephala* (Fabricius, 1784) (6.59, 95% CI: 6.26–6.94%), *Aldrichina grahami* (Aldrich, 1930) (3.06, 95% CI: 2.83–3.30%), *L. cuprina* (Wiedemann, 1830) (3.04, 95% CI: 2.82–3.28%), *L. illustris* (Meigen, 1826) (2.57, 95% CI: 2.36–2.79%), and *Fannia prisca* Stein (54) (1.46, 95% CI: 1.31–1.64%). A few other species were also present, but at very low numbers. The fly density increased from April, peaking in July, and then gradually decreased. The highest fly density was found in farmers’ markets (4.04 flies per trap), followed by green belts (3.04 per trap), outdoor environments of catering areas (2.62 flies per trap), and urban residential areas (2.55 flies per trap). Among the different cities, Hangzhou had the highest fly density (5.36 flies per trap), followed by Taizhou (4.44 flies per trap) and Zhoushan (4.01 flies per trap). The cities with the lowest fly densities were Ningbo (1.64 flies per trap), Huzhou (1.76 flies per trap), and Shaoxing (1.77 flies per trap) (Figure 2).

### 3.4 Cockroach density surveillance

**Figure 3:**
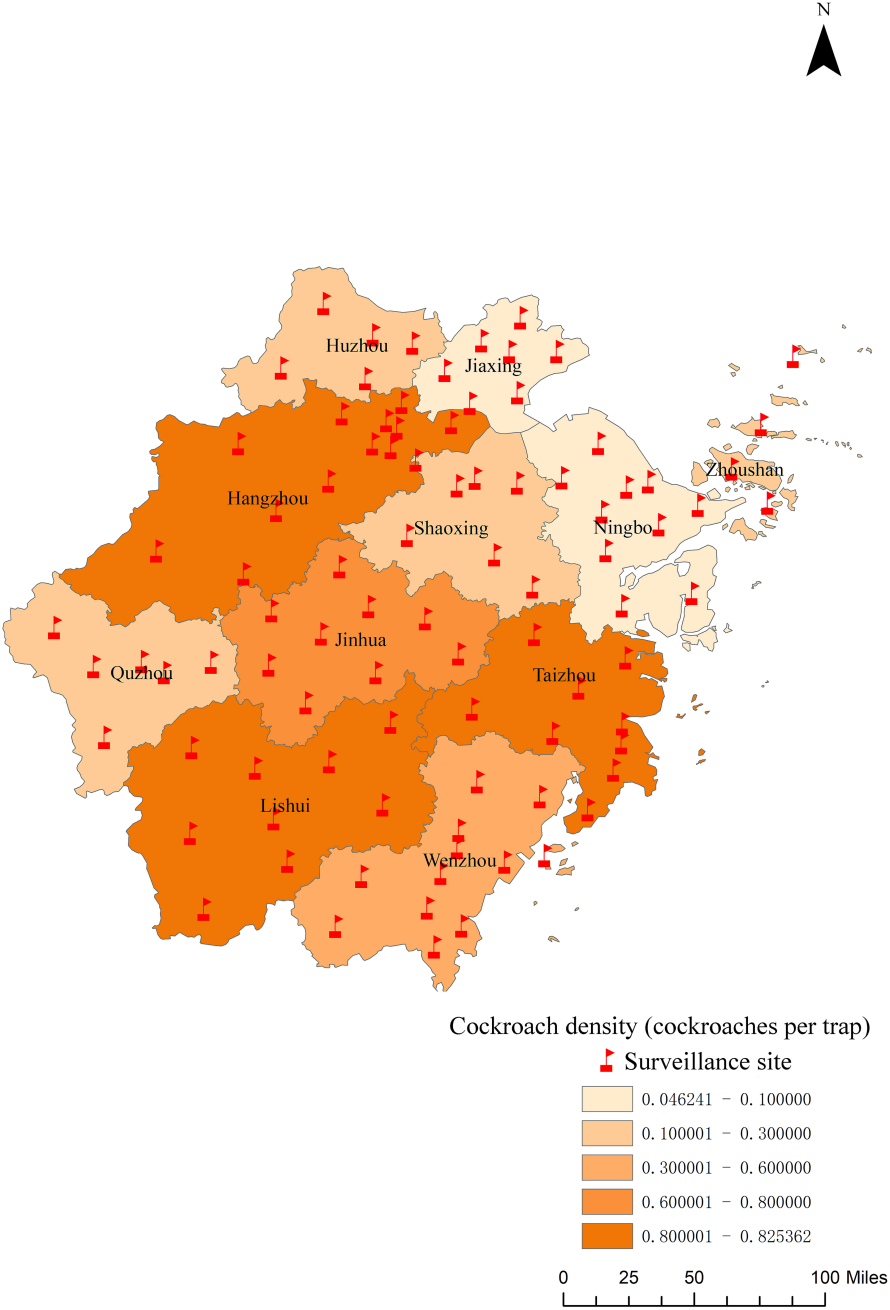
The regional distribution of cockroach density in Zhejiang Province in 2024.

A total of 77,564 sticky traps were set up across the province in 2024, in which 34,442 cockroaches were captured, representing a density of 0.44 ± 0.31 cockroaches per trap (Supplementary Table 3). *Blattella germanica* (Linnaeus, 1767) was the dominant species, with a total number of 33,453, accounting for 97.13% (95% CI: 96.95–97.30%) of the total catch. This was followed by *Periplaneta fuliginosa* (55) (1.38, 95% CI: 1.26–1.51%), *P. americana* (Linnaeus, 1758) (1.35, 95% CI: 1.24–1.48%), and *P. australasiae* (Fabricius, 1775) (0.08, 95% CI: 0.06–0.12%). The cockroach density showed an increasing trend throughout the year, peaking in November. Among the surveyed habitats, the highest cockroach density was found in farmers’ markets (1.75 cockroaches per trap), while the lowest was observed in hospitals (0.04 cockroaches per trap). Regarding cities, Hangzhou had the highest cockroach density (0.83 cockroaches per trap), followed by Lishui and Taizhou, both with densities of 0.82 cockroaches per trap. Ningbo and Jiaxing had the lowest densities, with values of 0.05 and 0.08 cockroaches per trap, respectively (Figure 3).

### 3.5 Tick density surveillance

For questing tick surveillance, a total of 92,134 meters of vegetation were sampled using the dragging method, resulting in the capture of 325 ticks (0.35 ± 1.32 ticks per flag per 100 m). *Haemaphysalis longicornis* Neumann (56) was the dominant species, accounting for 93.23% (95% CI: 89.96–95.49%) of the total capture, followed by *Ixodes sinensis* Teng, 1977 (5.23, 95% CI: 3.29–8.22%) and *Rhinpicephalus sanguineus* (Latreille, 1806) (0.62, 95% CI: 0.17–2.22%). The maximum density of the questing ticks was in March. Of the two habitats monitored, the tick density was higher in rural outdoor environments (0.64 ticks per flag per 100 m) than in scenic habitats (0.05 ticks per flag per 100 m). Among the different cities, Taizhou had the highest tick density (4.55 ticks per flag per 100 m), followed by Zhoushan city (1.27 ticks per flag per 100 m). In regions such as Hangzhou, Huzhou, Jiaxing, Jinhua, Lishui, Quzhou, and Shaoxing cities, either no questing ticks were captured, or the tick density was below 0.01 ticks per flag per 100 m (Supplementary Table 4).

**Figure 4:**
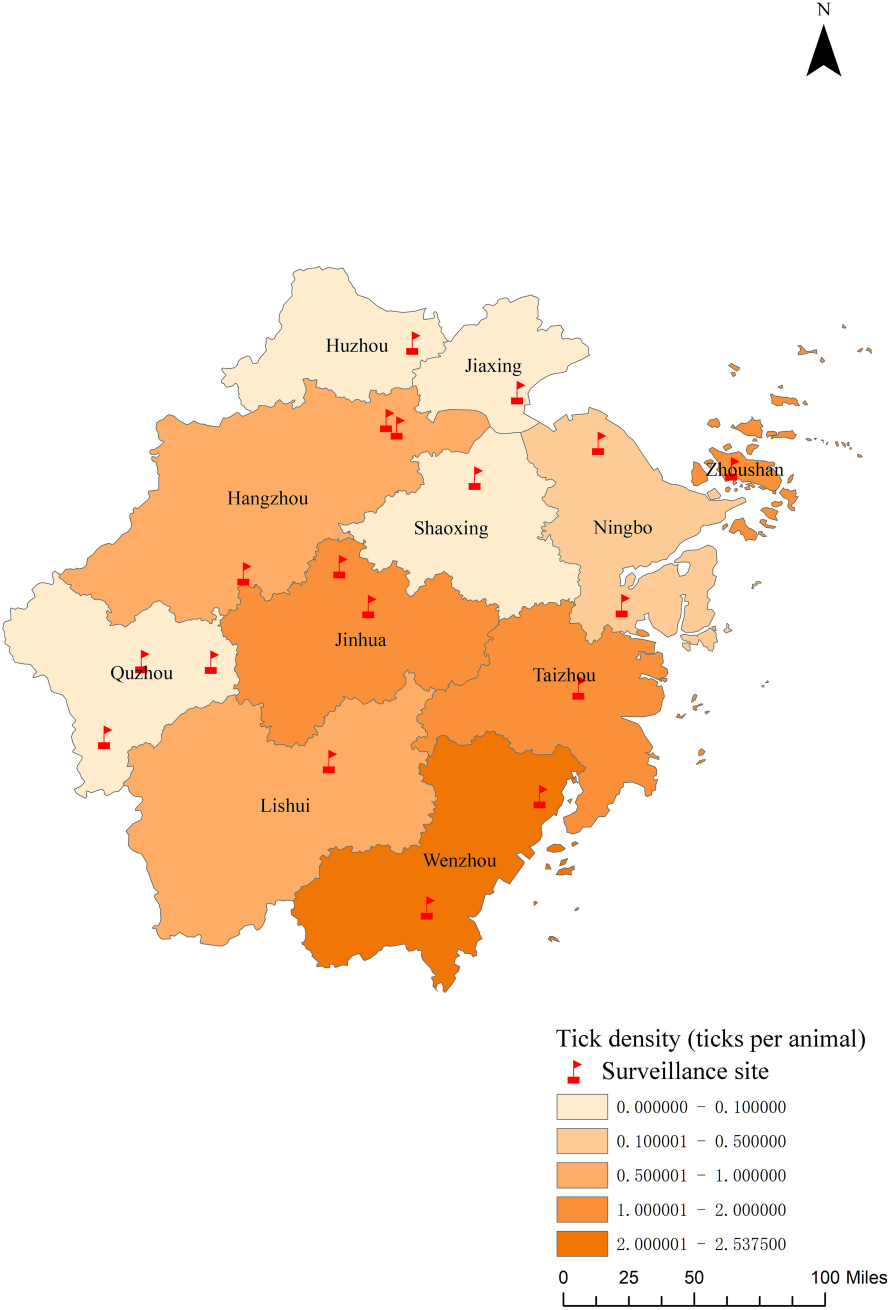
The regional distribution of parasitic tick density in Zhejiang Province in 2024.

A total of 1,500 animals were inspected for ticks in 2024, of which 151 showed infestation. A total of 760 ticks were collected, yielding an average intensity of 0.51 ± 0.81 ticks per animal. Ticks collected from the infested animals included *H. longicornis* (58.03, 95% CI: 54.49–61.49%), *R. sanguineus* (31.32, 95% CI: 28.12–34.70%), *R. microplus* (Canestrini, 1888) (7.76, 95% CI: 6.07–9.89%), and a few individuals of other tick species, such as *R. haemaphysaloides* (Supino, 1897) (1.05, 95% CI: 0.53–2.06%) and *I. sinensis* (0.92, 95% CI: 0.45–1.89%) (Supplementary Table 5.1). Among the different host animals examined, cattle and sheep displayed the highest tick densities (1.90 and 0.92 ticks per animal, respectively), followed by rural dogs (0.40 ticks per animal) and urban dogs (0.20 ticks per animal) (Supplementary Table 5.2). The maximum density of ticks collected from animals was in July. Among the cities, Wenzhou had the highest tick index (2.54 ticks per animal), followed by Zhoushan (1.63 ticks per animal), Taizhou city (1.53 ticks per animal), and Jinhua city (1.28 ticks per animal) (Figure 4).

### 3.6 Bedbug and chigger mite surveillance

A total of 4,360 rooms were surveyed for bedbugs in Zhejiang Province, including 1,230 in Hangzhou City, 1,240 in Ningbo City, 600 in Jinhua City, and 1,290 in Quzhou City. None of the rooms tested positive for bedbugs, and no signs of infestation were detected (Supplementary Table 6). A total of 90 rodents were captured for chigger mite monitoring, with 64 found to be positive, resulting in an infestation rate of 71.11% (95% CI: 61.04–79.46%). Overall, 1,055 chigger mites were detected, representing an index of 11.72. Of these, 515 chigger mites were identified, including 212 belonging to multiple species of *Walchia* Ewing, 1931 and 303 from the genus *Leptotrombidium* Nagayo et al. (58). For the different habitats, the mite infestation rate was highest in orchards, reaching 80.00% (95% CI: 37.55–98.97%), followed by wasteland/scrub (74.36, 95% CI: 58.92–85.43%), farmland (71.43, 95% CI: 52.94–84.75%), and rural residential areas (61.11, 95% CI: 38.62–79.69%) (Supplementary Table 7).

### 3.7 Rodent and mosquito pathogen evaluation

In our study, a survey of mosquito pathogens was conducted in five cities—Hangzhou, Ningbo, Quzhou, Taizhou, and Huzhou. A total of 27,402 female mosquitoes were tested, including 10,344 *Ae. albopictus*, 12,606 *Cx. pip. Pallens*, 2,580 *Cx. tritaeniorhynchus*, and 1,872 *An. sinensis*. Depending on the mosquito species, the pathogens tested included dengue virus, yellow fever virus, Japanese encephalitis virus, West Nile virus, Zika virus, and chikungunya virus, all of which returned negative results.

The presence of rodent pathogens was investigated in five cities—Hangzhou, Ningbo, Jinhua, Zhoushan, and Lishui. In total, 1,034 rodents were tested for Hantavirus, with 25 samples found to be positive (positivity rate: 2.42, 95% CI: 1.64–3.54%, Seoul virus (SEOV)). These infected rodents were from Zhoushan and Hangzhou (positivity rates of 10.84, 95% CI: 7.27–15.86%, and 1.50, 95% CI: 0.51–4.32%, respectively). A total of 985 rodents were tested for *L. interrogans*, yielding a positivity rate of 10.46% (95% CI: 8.70–12.52%). *Leptospira*-positive rodents were found in all the cities monitored, with the highest positivity rate being recorded in Lishui (17.41, 95% CI: 13.01–22.91%) and Zhoushan (14.78, 95% CI: 10.55–20.31%). Out of 630 rodent samples tested for *O. tsutsugamushi*, only one was positive (Ningbo), with an overall positivity rate of 0.16% (95% CI: 0.01–0.89%). None of the tested rodent samples showed positivity for Dabie Bandavirus or *R. typhi* (Table 5).

**Table 5:** The results of the pathogen investigation in rodents from Zhejiang Province in 2024.

Region	Hantavirus			Dabie Bandavirus			*Leptospira interrogans*			*Rickettsia typhi*			*Orientia tsutsugamushi*		
	No. of rodents	No. of positive tests	Positivity rate (%)	No. of rodents	No. of positivetests	Positivity rate (%)	No. of rodents	No. of positivetests	Positivity rate (%)	No. of rodents	No. of positivetests	Positivity rate (%)	No. of rodents	No. of positivetests	Positivity rate (%)
Hangzhou	200	3	1.50	200	0	0	200	16	8.00	0	0	–	200	0	00.00
Ningbo	200	0	0	200	0	0	200	5	2.50	0	0	–	200	1	0.50
Jinhua	207	0	0	207	0	0	158	13	8.23	25	0	0	25	0	00.00
Zhoushan	203	22	10.84	203	0	0	203	30	14.78	203	0	0	0	0	–
Lishui	224	0	0	255	0	0	224	39	17.41	161	0	0	205	0	00.00
Total (all regions)	1,034	25	2.42	1,065	0	0	985	103	10.46	389	0	0	630	1	0.16

## 4 Discussion

In our study, an integrated surveillance approach was employed to investigate the population density of important vector species and host animals, as well as to examine the relevant pathogen infections. Results found that *Cx. tritaeniorhynchus* and *Cx. pip. Pallens* were dominant mosquitoes particularly in livestock sheds, while *Ae. albopictus* larvae density was also high. Previous entomological surveys (18) have established that *Culex* spp. and *Ae. albopictus* represent the predominant mosquito taxa in Zhejiang Province. However, their distinct ecological and behavioral characteristics preclude the use of a single standardized surveillance method for simultaneous population assessment. Specifically, *Ae. albopictus* exhibits diurnal container-breeding habits, whereas *Culex* mosquitoes prefer nocturnal, open-water habitats. Given these constraints, we implemented a dual-approach monitoring strategy: the larval pipette method for *Aedes* surveillance and the light trap method for *Culex* population monitoring. This combination was selected to optimize both operational feasibility and data reliability, leveraging the larval pipette method’s proven sensitivity for detecting container-breeding *Aedes* immature stages while utilizing light traps to effectively capture host-seeking *Culex* adults during peak nocturnal activity periods. We found that the average BI was 12.97 in the surveyed cities, indicating that the risk of dengue or chikungunya transmission was moderate. *Ae. albopictus* is an important vector for dengue virus transmission in Zhejiang Province (20), especially considering the ongoing importation of dengue cases (21). Studies have established the BI as an important early warning indicator for dengue fever, with a BI value of 5 serving as the lowest threshold. Control measures are warranted if the BI exceeds 5 alongside reported dengue cases or if it exceeds 20 even in the absence of any dengue case (22). These *Aedes* density thresholds are also applicable to chikungunya fever surveillance and control, as integrated vector management strategies targeting *Aedes* mosquitoes are effective across multiple arboviruses transmitted by the same vector species (23). Discarded tires, natural water accumulations, and other containers had the highest BI values, so larval control should prioritize eliminating these high-risk habitats for maximum efficiency. In the adult mosquito surveillance using the light-trap method, *Cx. tritaeniorhynchus* and *Cx. pip. Pallens* were identified as the dominant species. However, as *Ae. albopictus* is not sensitive to light trap collection (18), the results likely do not accurately reflect its true population density. Notably, the highest mosquito density—81.07 mosquitoes per trap-night—was found in livestock sheds, with the majority of the catch being *Cx. tritaeniorhynchus*. This mosquito species is an important vector for the transmission of Japanese encephalitis (JE) (24). Deng X et al. found that Japanese encephalitis virus (JEV) strains isolated from mosquitoes during 2015–2018 all belonged to Genotype I with infection rates of 1.56, 2.36, 5.65 and 1.77 per 1,000 mosquitoes, respectively. Zhejiang Province was at a high risk of JE exposure due to relatively lower neutralizing antibody levels among the younger-aged population and higher infection rates of JEV in mosquitoes (25). As the livestock sheds are predominantly located in rural areas of Zhejiang Province, where many farmers have limited awareness of disease protection, the observed abnormally high density of *Cx. tritaeniorhynchus* in these habitats merits special attention, especially in areas with the highest mosquito densities, such as Taizhou and Huzhou. Pathogen testing was conducted on a large number of mosquitoes, encompassing major mosquito species such as *Cx. tritaeniorhynchus*, *Cx. pip. Pallens*, *Ae. albopictus*, and *An. sinensis*. However, no positive results were detected in our study. In 2020, Wu et al. tested for pathogens in 10,200 female mosquitoes in Zhejiang Province and also reported negative results (18). These consistent negative findings might be attributable to a low infection rate among the mosquito population, pathogen loads below assay detection limits, or pathogen presence in unsampled micro-foci. However, from a One Health perspective, entomological surveillance alone offers an incomplete picture. As Park & Lee emphasize, effective JE control necessitates coordinated efforts across veterinary, environmental, and human health sectors (26). Such integrated strategies enhance anticipation of outbreak seasons, optimize resource utilization, and provide the comprehensive inter-sectoral evidence required for accurate forecasting and prevention of JEV transmission.

In our study, the average rodent density was found to be 0.34 rodents per 100 trap-nights in 2024, which was relatively lower than the density of 0.58 rodents per 100 trap-nights reported for Zhejiang Province between 2017 to 2022 (17), indicating that rodent control in the province had achieved notable success. Rodent activity was sustained year-round, exhibiting a subtle bimodal peak in May and November, consistent with earlier findings (27). The presence of these two peaks may be attributed to both the influence of the rodent breeding season and the relative accessibility of food sources during these periods. *R. norvegicus* was the dominant rodent species in Zhejiang Province, followed by *S. murinus*, *M. musculus*, and *R. flavipectus*. These species are known to act as reservoir hosts for diseases such as Hemorrhagic Fever with Renal Syndrome (HFRS) and Leptospirosis (17). Zhejiang Province is recognized as a major endemic area for leptospirosis in China, with case numbers having risen significantly from 2010 to 2022 (28, 29). In our study, *L. interrogans* was detected in rodent samples from all monitored cities, with an overall positivity rate of 10.46%. Notably, the positivity rates in rodent samples from Lishui City and Zhoushan City exceeded 10%. These results revealed that *L. interrogans* was widespread among rodents in Zhejiang Province, highlighting an emerging public health threat that demands immediate attention. In addition to leptospirosis, a total of 4,240 cases of HFRS were reported in Zhejiang Province from 2009 to 2018, with cases documented in 80% of its counties (30). In our study, Hantavirus was detected in rodents from both Zhoushan and Hangzhou, indicating that HFRS still poses a transmission risk in Zhejiang Province. Furthermore, *O. tsutsugamushi*, the causative agent of scrub typhus, has a wide distribution range in China, including Zhejiang Province (31). Notably, 2,678 cases of scrub typhus were reported in Zhejiang Province from January 2016 to September 2023, resulting in an overall case fatality rate of 0.04% (32). In this work, one rodent sample from Ningbo city tested positive for *O. tsutsugamushi*, a finding that warrants close attention. Chigger mites are the exclusive vectors of scrub typhus. In Lishui city, the chigger mite infestation rate among the rodents inspected was 71.11%, representing an extremely high level. In all the monitored habitats, including orchards, wasteland/scrub, farmland, and rural residential areas, the infestation rates of mites on their rodent hosts’ bodies exceeded 60% in all cases, indicating a significant risk for human exposure to chigger mites and subsequent scrub typhus transmission.

Flies can serve as vectors for pathogen transmission among nonhuman primates, livestock, and humans (33). Indeed, some fly species, such as *L. sericata*, are used by forensic entomologists to estimate the minimum postmortem interval (34). Our study revealed that sarcophagid flies were the most dominant fly species, followed by *M. domestica* and *L. sericata*. This pattern may be linked to factors such as delayed garbage disposal, humus accumulation in green belts, and the high abundance of decaying animal matter resulting from the prevalence of seafood in coastal cities. The strong reproductive ability, adaptability, and resistance to insecticides of *B. germanica* have contributed to its emergence as a global issue (35). One study showed that the global mean infestation prevalence of *B. germanica* in human dwellings ranges from 40.0 to 70.0% (36), and continues to increase. In our investigation, *B. germanica* was the absolute dominant cockroach species in Zhejiang Province, accounting for 97.13% of the total catch. Therefore, the control of cockroaches in Zhejiang Province is essentially synonymous with the control of *B. germanica*. Among the various cities sampled, Hangzhou and Taizhou had higher densities of both cockroaches and flies, a finding that requires special attention. Concerning the different habitats, the highest densities of both cockroaches and flies were detected in farmers’ markets, potentially attributable to the warm and humid environment, abundance of food sources, and high levels of human and goods traffic that characterize these locations. Bedbugs constitute an important group of obligate hematophagous urban insect pests. Their resurgence over the past two decades is a global phenomenon, mainly driven by the development of insecticide resistance, increased global travel, and poor pest management practices (37). In the present study, bedbug density was monitored in key habitats across four cities, with over 4,000 rooms being inspected. No evidence of the presence of bedbugs or signs of bedbug infestation was observed in any of these rooms, indicating that the bedbug infestation level in Zhejiang Province is extremely low. However, given that bedbugs are still present in neighboring areas (38) and can be easily spread, ongoing monitoring of bedbugs remains essential.

*H. longicornis* was the major tick species identified in Zhejiang Province, followed by *R. sanguineus*. Notably, *H. longicornis* accounted for 93.23% of the total questing tick catch. Given its high density and wide distribution in China, *H. longicornis* is considered the primary vector for Dabie Bandavirus in the country (39). Because of the widespread presence of ticks, Zhejiang Province represents a natural epidemic focus of Severe Fever with Thrombocytopenia Syndrome (SFTS). It has been shown that the risk of SFTS occurrence increases with increasing tick density, with a generated response curve indicating that the risk was greater than 0.5 at tick densities exceeding 1.4 ticks per flag per 100 m (15). In our study, Taizhou city had the highest tick density at 4.55 ticks per flag per 100 m, which greatly surpassed this threshold. In addition to the presence of ticks, activities such as raising domestic animals, grazing, farming, contact with wild animals, and the presence of host animals are all risk factors for SFTS (17). Because such activities can amplify the chance of encountering ticks, we also screened host animals for infestation. Our results demonstrated that Wenzhou, Zhoushan, Taizhou, and Jinhua all had a tick density >1 tick per animal. Cattle and sheep, which have extremely frequent contact with humans, had the highest tick index, with the majority captured being *H. longicornis*. The areas with the highest tick densities in eastern Zhejiang Province coincided with those previously reported to have the highest SFTS incidences (40, 41), suggesting that tick density surveillance could play an important role in SFTS monitoring and early warning.

This research had several strengths. Our study was distinguished by its implementation of a province-wide integrated surveillance framework that simultaneously monitored both vectors and animal hosts, as well as selected associated pathogens. The findings provide an unparalleled evidence base for precision vector control and proactive early warning of emerging zoonoses. Despite these strengths, some limitations of this study should also be acknowledged. While we have established a comprehensive surveillance system, we did not conduct detailed analysis and discussion on the pathogen carrying status of key vectors such as ticks and chigger mites. Given that these vectors are significant carriers and transmitters of various zoonotic pathogens, the lack of detailed exploration in this regard may lead to an incomplete understanding of the overall epidemiological landscape in the study area. Besides, our analysis focused exclusively on the ecological and pathogen survey of vectors and reservoir hosts. Other critical environmental determinants, such as meteorological variables, population density, and socio-economic status, were not included and will need to be explored in future studies.

In conclusion, using an integrated surveillance approach, we identified the predominant vector and reservoir species in Zhejiang Province, including mosquitoes, flies, rodents, cockroaches, ticks, chigger mites, and bedbugs. Additionally, we identified the regions, seasons, and habitats where these species reach peak abundance, along with the pathogen carriage status of key vector mosquitoes and rodent hosts. These findings provide valuable information for the management of important vector and host animals, providing basic data for the prevention and control of VBDs from a public health perspective.

## Data Availability

The raw data supporting the conclusions of this article will be made available by the authors, without undue reservation.

## Supplementary material

SUPPLEMENTARY TABLE 1 Mosquito larvae surveillance results, Zhejiang Province, 2024.

SUPPLEMENTARY TABLE 2 Fly surveillance results, Zhejiang Province, 2024.

SUPPLEMENTARY TABLE 3 Cockroach surveillance results, Zhejiang Province, 2024.

SUPPLEMENTARY TABLE 4 Tick surveillance results (flagging method), Zhejiang Province, 2024.

SUPPLEMENTARY TABLE 5.1 Tick surveillance results (tick-picking method) in different cities, Zhejiang Province, 2024.

SUPPLEMENTARY TABLE 5.2 Tick surveillance results (tick-picking method) in different animals, Zhejiang Province, 2024.

SUPPLEMENTARY TABLE 6 Bedbug surveillance results, Zhejiang Province, 2024.

SUPPLEMENTARY TABLE 7 Chigger mite surveillance results, Zhejiang Province, 2024.
